# Antioxidant and Pro-Oxidant Properties of *Carthamus Tinctorius*, Hydroxy Safflor Yellow A, and Safflor Yellow A

**DOI:** 10.3390/antiox9020119

**Published:** 2020-01-29

**Authors:** Tiziana Bacchetti, Camilla Morresi, Luisa Bellachioma, Gianna Ferretti

**Affiliations:** 1 Department of Life and Environmental Sciences, Polytechnic University of Marche, 60131 Ancona, Italy; t.bacchetti@univpm.it (T.B.); m.morresi@libero.it (C.M.); luisabellachioma@gmail.com (L.B.); 2 Department of Clinical Sciences, Polytechnic University of Marche, 60131 Ancona, Italy

**Keywords:** safflower, HSYA, SYA, oxidative stress, lipid peroxidation

## Abstract

(1) *Carthamus Tinctorius L*. (safflower) is extensively used in traditional herbal medicine. (2) The aim of this study was to investigate the bioactive properties of polyphenol extracts from flowers of *Carthamus Tinctorius* (CT) cultivated in Italy. We also evaluated the properties of two bioactive water-soluble flavonoid compounds, hydroxy safflor yellow A (HSYA) and safflor yellow A (SYA), contained in *Carthamus Tinctorius* petals. (3) The total polyphenol content was 3.5 ± 0.2 g gallic acid equivalent (GAE)/100 g, flavonoids content was 330 ± 23 mg catechin equivalent (CE)/100 g in the flowers. The extract showed a high antioxidant activity evaluated by oxygen radical absorbance capacity (ORAC) and 2-diphenyl-1-picrylhydrazyl (DPPH) radical-scavenging assays. In addition, flower extract, SYA, and HSYA were able to reduce the susceptibility of low-density lipoprotein to copper-induced lipid peroxidation. In order to investigate the bioactive properties of flower extract, SYA, and HSYA we also studied their modulatory effect of oxidative stress on human dermal fibroblasts (HuDe) oxidized by tert-butyl hydroperoxide (*t*-BOOH). The CT extract at concentrations ranging from 0.01–20 μg GAE/mL of polyphenols, exerted a protective effect against *t*-BOOH triggered oxidative stress. At higher concentration the extract exerted a pro-oxidant effect. Similar results have been obtained using HSYA and SYA. (4) These results demonstrate a biphasic effect exerted by HSYA, SYA, and flower extracts on oxidative stress.

## 1. Introduction

Previous studies have demonstrated healthy promoting properties of seed of *Carthamus tinctorius L.* (safflower), a traditional herbal medicine used across Asia [1,2,3,4]. *Carthamus tinctorius L.* seeds are known as a source of α-linoleic acid and have been used to obtain cooking oil in Europe [4]. The word tinctorius essentially means ‘for dyeing’ in English. The flowers of safflower have been used historically as a colorant in food and as dye in the clothing industry. Bioactive compounds such as polyphenols account for the colour and taste of safflower [2]. The phenolic composition and antioxidant activity of safflower seeds and petals has been previously studied [4,5]. Gallic acid was the most abundant phenolic acid in *Carthamus tinctorius L.* flowers, other phenolic compounds are chlorogenic acid, syringic acid, quercetin-3-galactoside, and epicatechin [5]. Moreover, quinochalcone C-glycosides have been described in safflower, the most representative are the water soluble compounds hydroxy safflor yellow A (HSYA), safflor yellow A (SYA), and hydroxy safflor yellow B [4,5,6]. The bioactive properties of HSYA, used in Chinese medicine for treatment of cerebrovascular and cardiovascular disease, have been previously studied [7,8,9,10]. Anti-inflammatory properties [8] and neuroprotective effects have been reported [10]. Among molecular mechanisms, antioxidant activity has been described [7,11]. Moreover, HSYA significantly inhibits abnormal proliferation of tumor cell in the culture, without affecting normal endothelial cell growth [12]. HSYA reduces also apoptosis in pancreatic β-cells by attenuating oxidative damage and JNK/c-Jun signaling pathway [13]. Previous studies have shown that also SYA exerts an inhibitory effect of oxidative stress and apoptosis [14]. 

The aim of this study was to investigate the functional properties of extracts obtained by the flowers of *Carthamus Tinctorius L.* cultivated in Italy. We evaluated polyphenol and flavonoid levels and antioxidant properties using different experimental approaches. Recent studies on cells in the culture, demonstrate a biphasic effect exerted by several polyphenols on oxidative stress, acting as antioxidants at low concentrations but pro-oxidant at higher concentrations [15,16]. In order to investigate whether safflower extracts also exert a biphasic effect on oxidative stress, we used HuDe cells as an experimental model. The modulatory effect of HSYA and SYA on oxidative stress, was also studied. 

## 2. Materials and Methods 

### 2.1. Reagents

All cell culture reagents were obtained by Euroclone (Euroclone, Italy). All chemical reagents were obtained by Sigma Aldrich (Sigma, St Louis, MO, USA). HSYA and SYA compounds were purchased from Chem Faces (CheCheng Rd., Wuhan Economic and Technological Development Zone, Wuhan, Hubei 430056, China). The fluorescent probe 2’,7’-dichlorodihydrofluorescein diacetate (H_2_DCFDA) (C400) was supplied by Invitrogen (Invitrogen, Carlsbad, CA, USA).

### 2.2. Preparation of Extract

*Carthamus Tinctorius* (CT) was kindly supplied by Terra e Vita, Recanati (MC), Italy. The flowers were washed and the petals were dried in an oven for 5 h at 40 °C. Five grams of the powdered sample was incubated with 100 mL ultra-filtered water at 80 °C for 10 min in a water bath shaker.

Subsequently after centrifugation (10 min at 1000 *g*), the supernatant was filtered with 0.22 μm filters, 30 mm diameter [17].

### 2.3. Total Phenolic Composition

Total phenolic content (TPC) was evaluated following the Folin–Ciocalteu assay [18]. Briefly, 20 µL of extract were used. 0.1 mL of Folin–Ciocalteu phenol reagent and 0.3 mL sodium carbonate solution (20%) were added to tubes. After incubation for 40 min at 37 °C, absorbance was evaluated at 765 nm using a double-beam UV–Vis spectrophotometer (Shimadzu UV-2401 PC - Kyoto 604-8511 Shimadzu Corporation, Kyoyo, Japan). Blank samples were prepared using 20 μL of water and treated as described above. Gallic acid (GA) was used to develop a 0.1–1.3 mg/mL standard curve. The experiments were carried out in triplicate and TPC are expressed as milligrams of GA equivalent per 100 g of dry weight (mg GAE/100 g dw).

### 2.4. Total Flavonoids Evaluation

Total flavonoid content (TFC) was quantified according to the method of Kim et al. [19]. Briefly, 500 µL of extracts were used. 150 µL of NaNO_2_ (5%) was added to tubes. At the end of incubation (10 min) at room temperature, 150 µL of 10% AlCl_3_ were added and samples were further incubated for 10 min at room temperature. After that, 2 mL of NaOH (4%) were added. After incubation for 15 min, absorbance was evaluated at 415 nm against the blank using a double-beam UV–Vis spectrophotometer. Catechin was used as standard for the calibration curve (0–150 µg/mL). All the experiments were done in triplicate and the results are expressed as milligrams of catechin equivalent (CE) of 100 g of dry weight (mg CE/100 g dw).

### 2.5. Antioxidant Activity 

#### 2.5.1. Oxygen Radical Absorbance Capacity (ORAC) Assay

The antioxidant activity of CT extracts or HSYA and SYA was determined using an oxygen radical absorbance capacity (ORAC) assay [20]. This assay is based on the ability of the antioxidants to prevent loss of fluorescence signal of the fluorescent probe Fluorescein by scavenging peroxyl radicals generated by thermal decomposition of 2,2′-azobis (2-methylpropionamide) dihydrochloride (AAPH). The reaction was carried out in 75 mM sodium phosphate buffer (pH 7.4) and the final reaction mixture was 200 µL. 150 µL of 0.08 mM Fluorescein and 25 µl of CT extract, HSYA, and SYA were added to each well of a 96-well black polystyrene microplate. The mixture was preincubated for 10 min at 37 °C before adding 25 µL of 150 mM AAPH solution, using a multichannel pipette.

Fluorescence emission intensity was recorded every 5 min for 3 h at λ ex 485/λ em 530 nm in a Multi-Mode Microplate Reader Synergy^TM^ HT (BioTek Instruments, Inc., Winooski, VI, USA). A blank sample containing PBS, Fluorescein, and AAPH was prepared. Trolox (from 5 to 300 µM) was used to calibrate the assay. Samples were analyzed in triplicate. The final ORAC values were calculated using the net area under the decay curves (AUC).

Data about CT extract were expressed as Trolox equivalents (mmol TE/100 g dw). For SYA and HYSA we calculated the Trolox index as reported by Martinet et al. [21]:

Trolox index = Delta AUC _sample_ × [Trolox_concentration_]/Delta AUC _Trolox_ × [sample_concentration_] (1)

#### 2.5.2. 2-Diphenyl-1-picrylhydrazyl (DPPH) Radical-Scavenging Assay

The antioxidant activity of extracts was also determined using a 2,2-diphenyl-1-picrylhydrazyl (DPPH) radical-scavenging assay [22]. Aliquots of extracts (50 μL) were added to 1 mL of DPPH solution (5 mM) and the absorbance (ABS) was determined at 517 nm after 15 min of incubation. All samples were analyzed in triplicate. We calculated the percentage of scavenging and the inhibitory concentration (IC_50_). IC_50_ is the concentration of an antioxidant which exerts a 50% inhibition of free radical activity. EC_50_ (effective concentration) values and antiradical power (ARP) have been also evaluated:

Scavenging (%) = [(ABS_CTRL_ − ABS_sample_)/ABS_CTRL_] × 100 (2)

EC_50_ = IC_50_ (µg/mL)/DPPH (µg/mL) *t* = 0 (3)

ARP = 1/EC_50_ (4)

#### 2.5.3. Evaluation of the Formation of Conjugated Dienes 

Human low-density lipoproteins (LDL) were isolated from plasma by ultracentrifugation [23]. Lipid peroxidation and formation of conjugated dienes was followed monitoring changes in absorbance at 234 nm of LDL (1 mg/mL) during oxidative stress induced in vitro with copper (5 μM), in the absence or in the presence of CT extract. Increasing concentrations of HSYA and SYA (0–60 µg/mL) were used. The effects of safflower extracts were evaluated in the range of polyphenols (0–17 µg GAE/mL). The kinetic was studied for 4 h into 96-well plates. The lag time was calculated from the oxidation curve and percentage of inhibition rate was calculated:

Inhibition (%) = [(lag time_sample_ − lag time_CTRL_)/lag time _CTRL_] × 100 (5)

### 2.6. Cell Culture and Treatment 

HuDe cells were cultured in Dulbecco’s Modified Eagle Medium (DMEM) containing 10% (v/v) heat inactivated fetal bovine serum (FBS), 2 mM glutamine, 100 U/mL penicillin, 100 µg/ml streptomycin, 10 mM non-essential amino acids. Cells were grown at 37 °C in a humidified atmosphere containing 5% (v/v) CO_2_.

#### 2.6.1. Cell Viability 

The effect of safflower extract on cell viability was evaluated using the methyl thiazolyl tetrazolium (MTT) assay [24]. Briefly, HuDe cells were seeded at a density of 5 × 10^3^ cells/well into a 96-well plate and incubated at 37 °C in an atmosphere of 5% CO_2_. After plating overnight, 100 µL of CT aqueous extract containing increasing concentrations of polyphenols (0–150 µg GAE/mL), HSYA, or SYA (0–150 µg/mL) were added to the cell media. Cells were then incubated at 37 °C for 48 h. Then, 100 μL of MTT solution (5 mg/mL) was added to each well. The absorbance was measured at 540 nm with a Multi-Mode Microplate Reader Synergy^TM^ HT (BioTek Instruments, Inc.).

Moreover, we evaluated the effect of safflower extract, HSYA, or SYA on Lactate Dehydrogenase (LDH) release. LDH release, a good indicator of cellular damage, was measured with a commercially available LDH assay kit as previously described [25]. Absorbance was read at 340 nm in a Multi-Mode Microplate Reader Synergy^TM^ HT (BioTek Instruments, Inc.).

#### 2.6.2. Intracellular Reactive Oxygen Species (ROS) Levels

The formation of ROS in cells treated in different experimental conditions was evaluated using H_2_DCFDA as a probe [26]. H_2_DCFDA was dissolved in dimethyl sulfoxide (DMSO) as stock solution and kept frozen in −20 °C. Cells (25 × 10^3^ cells/well) were seeded 24 h before treatment with CT extract, HSYA, or SYA. After 48 h of treatment with CT extract, HSYA, or SYA at the same concentrations used in the test of viability, the medium was removed, and samples were washed with PBS. Then, cells were pre-treated for 45 min at 37 °C with the fluorescent probe 10 µM as final concentration. At the end of the incubation, H_2_DCFDA was removed and the fluorescence of the cells from each well was measured and recorded on a fluorescence plate reader at λ_ex_/λ_em_ (485/535 nm). Multi-Mode Microplate Reader Synergy^TM^ HT (BioTek Instruments, Inc.). The effect of CT extract, HSYA, or SYA was also studied in cells oxidized using 50 µM tert-butyl hydroperoxide (*t*-BOOH). 

### 2.7. Statistical Analysis 

The data from cell experiments are representative of five independent experiments and the data are shown as the mean ± SD. The Student’s *t*-test was applied and differences were considered to be significantly different if *p* < 0.05 (Origin, OriginLab Corporation, Northampton, MA, USA).

## 3. Results

### 3.1. Total Polyphenols, Total Flavonoids, and Antioxidant Properties 

Total polyphenols and total flavonoids in *Carthamus Tinctorius* (CT) were 3.5 ± 0.2 g GAE/100 g and 330 ± 23 mg CE/100 g, respectively. The total antioxidant activity of *Carthamus Tinctorius* evaluated by an ORAC assay was 130.2 ± 12.3 mmol TE/100 g. 

As shown in Table 1, Trolox index values were in the order HSYA > SYA.

Using DPPH, we obtained an IC_50_ value of 13.4 ± 1.0 μg GAE/mL for CT extract. IC_50_ and EC_50_ values were lower for HSYA compared with SYA (Table 2). ARP was in the order HSYA > safflower extract > SYA (Table 2).

### 3.2. Kinetics of LDL Oxidation 

**Figure 1:**
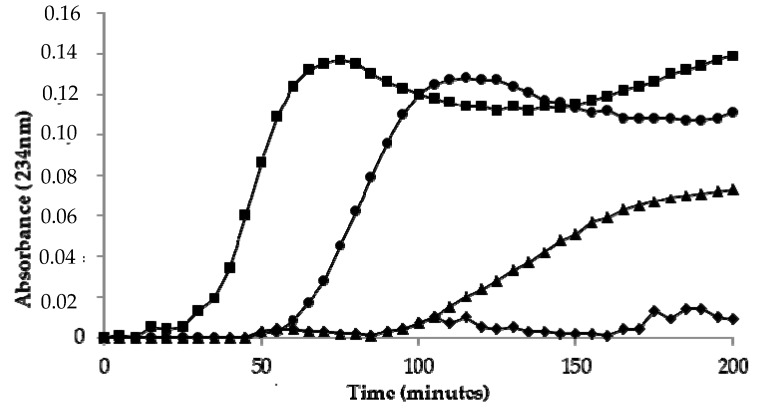
Kinetics of LDL oxidation by 5 µM CuSO_4_ as measured by the formation of conjugated dienes. Control LDL (◾), LDL incubated in the presence of *Carthamus Tinctorius* (CT) extract (17 µg gallic acid equivalent/mL (▲), or in the presence of 60 µg/mL of hydroxy safflor yellow A (HSYA) (⬥) or safflor yellow A (SYA) (●).

LDL oxidized by copper ions showed a lag time of 30 ± 2 min. A significant increase in lag time was observed in LDL oxidized in the presence of the CT extract (Table 3) (Figure 1). The protective effect was dependent on the concentration of polyphenols. A significant increase in lag time was also observed in LDL oxidized in the presence of HSYA or SYA (Table 3) (Figure 1).

### 3.3. Effects of Carthamus Tinctorius Extracts on Cells Viability and Intracellular ROS in HuDe Cells

**Figure 2:**
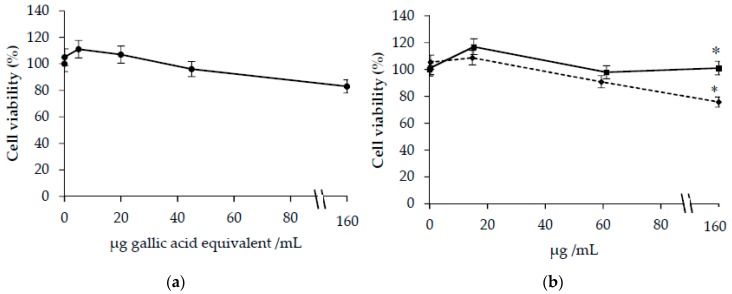
Viability in HuDe cells treated for 48 h in the presence of increasing concentrations of *Carthamus Tinctorius* (CT) extract (**a**) or hydroxy safflor yellow A (HSYA) (◾) and safflor yellow A (SYA) (⬥) (**b**). Phenolic concentrations of CT extract are expressed as µg gallic acid equivalent/mL. * *p* < 0.05 vs. cells incubated in the absence of CT extracts or SYA and HSYA.

As shown in Figure 2a, no significant modification in cell viability was observed in cells treated with CT extract at concentrations ranging from 0–150 µg GAE/mL.

No significant modifications in cellular viability were observed also in cells treated with HSYA. A decrease in cellular viability was demonstrated in cells incubated with SYA at the highest concentrations (150 µg/mL) (Figure 2b). The study of LDH release confirms these results (data not shown).

**Figure 3:**
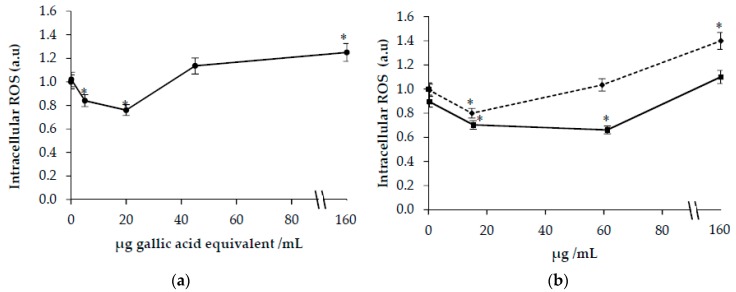
Intracellular reactive oxygen species (ROS) formation in HuDe cells treated for 48 h in the presence of increasing concentrations of *Carthamus Tinctorius* (CT) extract (**a**) or hydroxy safflor yellow A (HSYA) (◾) and safflor yellow A (SYA) (⬥) (**b**). Phenolic concentrations of CT extract are expressed as µg gallic acid equivalent/mL. * *p* < 0.05 vs. cells incubated in the absence of CT extracts or SYA and HSYA.

A significant decrease in ROS levels was observed in cells incubated with safflower extract (Figure 3a) at concentrations lower than 20 μg GAE/mL. On the contrary, an increase was observed at the highest concentration (150 μg GAE/mL) (Figure 3a). As shown in Figure 3b, a decrease in ROS levels was observed at concentrations of HSYA lower than 60 μg/mL. At the highest concentration of HSYA or SYA (150 μg/mL), a significant increase of ROS levels was observed.

### 3.4. Effects of Carthamus Tinctorius Extracts on ROS in t-BOOH-Treated Cells

**Figure 4:**
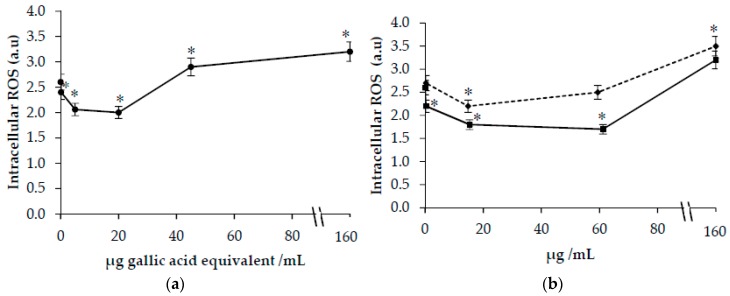
Intracellular ROS formation in HuDe cells treated with the pro-oxidant tert-butyl hydroperoxide (*t*-BOOH) (50 µM) in the presence of increasing concentrations of *Carthamus Tinctorius* (CT) extract (**a**) or hydroxy safflor yellow A (HSYA) (◾) and safflor yellow A (SYA) (⬥) (**b**). Phenolic concentrations of CT extract are expressed as µg gallic acid equivalent/mL. * *p* < 0.05 vs. cells treated with t-BOOH in the absence of CT extracts or SYA and HSYA.

ROS levels were higher in cells treated with 50 μM t-BOOH compared with control cells (2.6 ± 0.1 vs. 1.0 ± 0.1). As shown in Figure 4a, a protective effect against ROS formation was demonstrated in cells oxidized in the presence of low concentrations of CT extracts (0.1–20 μg GAE/mL). A protective effect against *t*-BOOH-induced ROS formation was observed also in cells treated with HSYA at concentrations ranging from 0.3 to 60 μg/mL. On the contrary, at the highest concentrations of safflower extract (150 μg GAE/mL), HSYA, or SYA (150 μg/mL) a pro-oxidant effect was demonstrated with a significant increase in intracellular levels of ROS compared with cells oxidized with 50 μM *t*-BOOH.

## 4. Discussion

The medicinal properties of safflower have been recently reviewed [2,3]. Among mechanisms potentially involved in the protective roles, both anti-inflammatory and antioxidative roles have been proposed. Our results confirmed that the water extracts obtained from the flowers of safflower contain polyphenols (about 3.5 g GAE/100 g) and flavonoids (about 330 mg CE/100 g), in agreement with literature [4,5]. A comparison with literature data demonstrates that ORAC values of safflower extracts are in the range observed in vegetables and spices [20]. The Trolox index was about three times higher for HSYA than SYA. HSYA had a Trolox index comparable to quercetin and kaempferol [21].

Using the DPPH assay, we demonstrated that safflower extracts exert scavenging properties. The comparison of the anti-radical activity parameters IC_50_, EC_50_, and antiradical power (ARP) of DPPH, demonstrates that HSYA exerted a higher scavenging property compared with SYA and CT extract. The values observed for HSYA are comparable to those reported in literature for gallic acid. 

To better investigate the modulatory properties of oxidative stress exerted by safflower extract, SYA, and HSYA, ex vivo methods were also used by assessing their ability to inhibit the lipid peroxidation of LDL induced in vitro by incubation with copper ions. The increase of levels of conjugate diene is widely used to investigate lipid peroxidation [27,28]. A significant increase of the lag phase was observed in LDL oxidized by copper ions in the presence of safflower extract. The effect was concentration dependent and demonstrated the ability of safflower extract to modulate the susceptibility of LDL to lipid peroxidation. The antioxidant effect against lipoprotein lipid peroxidation could be related to the ability of safflower polyphenols to exert a chelating effect or behave as “radical scavengers”. The results could be of physiological relevance, in fact oxidation of LDL occurs in vivo and oxidized LDL are involved in the development of atherosclerosis and other chronic-degenerative diseases [29]. Two serotonin derivatives, *N*-(*p*-coumaroyl) serotonin and *N*-feruloylserotonin and their glucoside derivatives have been identified as bioactive constituents of safflower extract [30]. It has been demonstrated that these serotonin derivatives are absorbed into circulation and protect against atherosclerotic lesion development [30]. Other polyphenols could be involved in the protective effect against lipid peroxidation of LDL. In fact, a protective effect against lipid peroxidation of LDL was exerted also by HSYA and SYA. Several polyphenols exert antioxidant properties in vitro and are of interest because they could help protect the human body against damages induced by reactive free radicals generated in human diseases such as atherosclerosis, Alzheimer’s disease, and even in the aging process [31,32]. 

A relationship between antioxidant activity and structural characteristics of polyphenols has been reported by several authors [33]. Literature data suggest that the scavenging activity of phenolic compounds is directly associated with the presence of hydroxyl groups [33]. It is, therefore, possible to suggest that the difference in antioxidant activity observed between HSYA and SYA could be likely related to the structural characteristics of the two bioactive compounds and, in particular, to the number of –OH groups and their position.

As a by-product of oxidative phosphorylation, a moderate quantity of ROS is necessary for cell survival and proliferation [34]. Previous studies have shown that HSYA treatment alleviates oxidative stress on neurons [10] and endothelial cells [9]. Even SYA inhibits cellular oxidative stress and apoptosis in cultured rat cardiomyocytes [14]. We confirmed that safflower extract and HSYA exert antioxidant properties as shown by the decrease of intracellular ROS levels in HuDe cells. The ability to modulate intracellular ROS levels was evaluated also in cells treated by tert-butyl hydroperoxide (*t*-BOOH). The effect on intracellular ROS formation was dependent on polyphenol concentration. At lower concentrations of polyphenols, a protective effect was observed suggesting that CT polyphenols may also play an antioxidant role at the cellular level. In contrast, higher CT polyphenol concentrations, HSYA or SYA increased intracellular ROS levels.

The results of this study support previous studies showing that polyphenols in relation to their concentration can have a biphasic effect. At low concentrations, polyphenols can act as antioxidants by acting as “radical scavengers” or by other mechanisms. Conversely, at higher concentrations, polyphenols can promote generation of ROS at a cellular level as previously suggested [15]. The modulatory biphasic effect of polyphenols on oxidative stress has been studied in different cell models [15]. The pro-oxidative and antioxidative properties of plant-derived antioxidant polyphenols depend on different factors as their metal-reducing potential, chelating behavior, and solubility characteristics [15,35]. Dual antioxidant and pro-oxidant activities have been demonstrated for several plant-derived polyphenols including phenolic acids (gallic acid, syringic acid, vanillic acid, ellagic acid, caffeic acid, coumaric acid, chlorogenic acid, ferulic acid), myricetin, quercetin, rutin, kaempferol, (+)-catechin, (−)-epicatechin, delphinidin, and malvidin [35].

## 5. Conclusions

In conclusion, although the phenolics inhibit oxidation in certain systems, this does not mean that they protect against all forms of oxidative damage. The evidence that dietary polyphenols exert protective properties against oxidative stress in some experimental models is strong. However, polyphenols can also exert pro-oxidative properties under certain conditions and in certain tissues [16]. Neither pro-oxidant nor antioxidant activities have yet been clearly established to occur in vivo in humans. Therefore, consumption of large amounts of polyphenols in the form of either foods or supplements might not be prudent until the bioactivity of these compounds is better understood.

## Figures and Tables

**Table 1:** Trolox index values for hydroxy safflor yellow A (HSYA) and safflor yellow A (SYA). * *p* < 0.005 vs. HSYA; ° Data from literature [21].

Compound	Trolox Index
**TROLOX**	1.0 ± 0.2
**HSYA**	7.1 ± 0.3
**SYA**	2.1 ± 0.1 *
**Quercetin** °	10.7
**Kaempferol** °	10.2
**Ferulic acid** °	3.5

**Table 2:** Comparison of values of antiradical activity evaluated by 2-diphenyl-1-picrylhydrazyl (DPPH) of *Carthamus Tinctorius* (CT) extracts, hydroxy safflor yellow A (HSYA) and safflor yellow A (SYA): IC_50_, EC_50_, and antiradical power (ARP). * *p* < 0.05 vs. CT extract; # *p* < 0.05 vs. HSYA.

Sample	IC_50_	EC_50_	ARP
**CT extract**	13.4 ± 1.0 (μg GAE/mL)	0.17 ± 0.01 (μg GAE/μg DPPH)	5.9 ± 0.2
**HSYA**	7.3 ± 1.2 * (μg HSYA/mL)	0.09 ± 0.01 * (μg HSYA/μg DPPH)	11.1 ± 0.8 *
**SYA**	30.3 ± 2.9 *^#^ (μg SYA/mL)	0.38 ± 0.06 *^#^ (μg SYA/μg DPPH)	2.6 ± 0.2 *^#^

**Table 3:** Lag time and percentage of inhibition of conjugated dienes formation in Cu^++^—triggered LDL oxidation in the absence or in the presence of increasing concentrations of *Carthamus Tinctorius* (CT) extracts, hydroxy safflor yellow A (HSYA) and safflor yellow A (SYA). Phenolic concentrations of CT extract are expressed as µg gallic acid equivalent (GAE)/mL. * *p* < 0.05 vs. LDL oxidized in the absence of polyphenols (control).

Sample	Concentration	Lag Time (Minutes)	Inhibition (%)
**Ctrl**	0	30 ± 2	-
**CT extract (µg GAE/mL)**	0.34	44 ± 3 *	46 ± 2
	1.70	59 ± 2 *	96 ± 1
	17.0	90 ± 4 *	200 ± 4
**HYSA (µg/mL)**	1.2	56 ± 6 *	86 ± 4
	6.0	65 ± 2 *	116 ± 1
	60.0	>200 *	-
**SYA (µg/mL)**	1.2	29 ± 5	0
	6.0	35 ± 6	16 ± 4
	60.0	49 ± 4 *	63 ± 2

## References

[1] Bukhari I.A. (2013). The central analgesic and anti-inflammatory activities of the methanolic extract of *Carthamus oxycantha*. J. Physiol. Pharmacol..

[2] Delshad E., Yousefi M., Sasannezhad P., Rakhshandeh H., Ayati Z. (2018). Medical uses of *Carthamus tinctorius* L. (Safflower): A comprehensive review from Traditional Medicine to Modern Medicine. Electron. Physician..

[3] Zhou X., Tang L., Xu Y., Zhou G., Wang Z. (2014). Towards a better understanding of medicinal uses of *Carthamus tinctorius* L. in traditional Chinese medicine: A phytochemical and pharmacological review. J. Ethnopharmacol..

[4] Kim E.O., Oh J.H., Lee S.K., Lee J.Y., Choi S.W. (2007). (*Carthamus tinctorius* L.) seeds. Antioxidant properties and quantification of phenolic compounds from safflower. Food Sci. Biotechnol..

[5] Salem N., Msaada K., Hamdaoui G., Limam F., Marzouk B. (2011). Variation in phenolic composition and antioxidant activity during flower development of safflower (*Carthamus tinctorius* L.). J. Agric. Food Chem..

[6] Jiang T.F., Lv Z.H., Wang Y.H. (2005). Separation and determination of chalcones from *Carthamus tinctorius* L. and its medicinal preparation by capillary zone electrophoresis. J. Sep. Sci..

[7] Chen L., Xiang Y., Kong L., Zhang X., Sun B., Wei X., Liu H. (2013). Hydroxysafflor yellow A protects against cerebral ischemia-reperfusion injury by anti-apoptotic effect through PI3K/Akt/GSK3beta pathway in rat. Neurochem. Res..

[8] Wang C.C., Choy C.S., Liu Y.H., Cheah K.P., Li J.S., Wang J.T., Yu W.Y., Lin C.W., Cheng H.W., Hu C.M. (2011). Protective effect of dried safflower petal aqueous extract and its main constituent, carthamus yellow, against lipopolysaccharide-induced inflammation in RAW264.7 macrophages. J. Sci. Food Agric..

[9] Wang J., Zhang Q., Xie H., Gu L.G., Niu X.Y., Liu L.T. (2009). Effect of hydroxy safflor Yellow A on the proliferation of human umbilical vein endothelial cells with the stimulus of tumor cell conditioned medium. CJTCMP.

[10] Zhu H., Wang Z., Ma C., Tian J., Fu F., Li C., Guo D., Roeder E., Liu K. (2003). Neuroprotective effects of hydroxysafflor yellow A: In vivo and in vitro studies. Planta Med..

[11] Wei X., Liu H., Sun X., Fu F., Zhang X., Wang J., An J., Ding H. (2005). Hydroxysafflor yellow A protects rat brains against ischemia-reperfusion injury by antioxidant action. Neurosci. Lett..

[12] Liu L., Si N., Ma Y., Ge D., Yu X., Fan A., Wang X., Hu J., Wei P., Ma L. (2018). Hydroxysafflor-Yellow A Induces Human Gastric Carcinoma BGC-823 Cell Apoptosis by Activating Peroxisome Proliferator-Activated Receptor Gamma (PPARgamma). Med. Sci. Monit..

[13] Zhao Y., Sun H., Li X., Zha Y., Hou W. (2018). Hydroxysafflor yellow A attenuates high glucose-induced pancreatic β-cells oxidative damage via inhibiting JNK/c-jun signaling pathway. Biochem. Biophys. Res. Commun..

[14] Duan J.L., Wang J.W., Guan Y., Yin Y., Wei G., Cui J., Zhou D., Zhu Y.R., Quan W., Xi M.M. (2013). Safflor yellow A protects neonatal rat cardiomyocytes against anoxia/reoxygenation injury in vitro. Acta Pharmacol. Sin..

[15] Halliwell B. (2008). Are polyphenols antioxidants or pro-oxidants? What do we learn from cell culture and in vivo studies?. Arch. Biochem. Biophys..

[16] Leon-Gonzalez A.J., Auger C., Schini-Kerth V.B. (2015). Pro-oxidant activity of polyphenols and its implication on cancer chemoprevention and chemotherapy. Biochemical. Pharmacol..

[17] Cai Y., Luo Q., Sun M., Corke H. (2004). Antioxidant activity and phenolic compounds of 112 traditional Chinese medicinal plants associated with anticancer. Life Sci..

[18] Ainsworth E.A., Gillespie K.M. (2007). Estimation of total phenolic content and other oxidation substrates in plant tissues using Folin–Ciocalteu reagent. Nat. Protoc..

[19] Kim D.O., Chun O.K., Kim Y.J., Moon H.Y., Lee C.Y. (2003). Quantification of polyphenolics and their antioxidant capacity in fresh plums. J. Agric. Food Chem..

[20] Gillespie K.M., Chae J.M., Ainsworth E.A. (2007). Rapid measurement of total antioxidant capacity in plants. Nat. Protoc..

[21] Martin I., Aspee A., Torres P., Lissi E., Lopez-Alarcon C. (2009). Influence of the target molecule on the oxygen radical absorbance capacity index: A comparison between alizarin red- and fluorescein-based methodologies. J. Med. Food.

[22] Brand-Williams W., Cuvelier M.E., Berset C.L.W.T. (1995). Use of a free radical method to evaluate antioxidant activity. Food Sci. Technol..

[23] Chung B.H., Segrest J.P., Ray M.J., Brunzell J.D., Hokanson J.E., Krauss R.M., Beaudrie K., Cone J.T. (1986). Single vertical spin density gradient ultracentrifugation. Methods Enzymol..

[24] Sladowski D., Steer S.J., Clothier R.H., Balls M. (1993). An improved MTT assay. J. Immunol. Methods.

[25] Decker T., Lohmann-Matthes M.L. (1988). A quick and simple method for the quantitation of lactate dehydrogenase release in measurements of cellular cytotoxicity and tumor necrosis factor (TNF) activity. J. Immunol. Methods.

[26] Wang H., Joseph J.A. (1999). Quantifying cellular oxidative stress by dichlorofluorescein assay using microplate reader. Free Radic. Biol. Med..

[27] Esterbauer H., Gebicki J., Puhl H., Jurgens G. (1992). The role of lipid peroxidation and antioxidants in oxidative modification of LDL. Free Radic. Biol. Med..

[28] Amarowicz R., Pegg R.B. (2017). The Potential Protective Effects of Phenolic Compounds against Low-density Lipoprotein Oxidation. Curr. Pharm. Des..

[29] Kattoor A.J., Pothineni N.V.K., Palagiri D., Mehta J.L. (2017). Oxidative Stress in Atherosclerosis. Curr. Atheroscler. Rep..

[30] Koyama N., Kuribayashi K., Seki T., Kobayashi K., Furuhata Y., Suzuki K., Arisaka H., Nakano T., Amino Y., Ishii K. (2006). Serotonin derivatives, major safflower (*Carthamus tinctorius* L.) seed antioxidants, inhibit low-density lipoprotein (LDL) oxidation and atherosclerosis in apolipoprotein E-deficient mice. J. Agric. Food Chem..

[31] Li A.N., Li S., Zhang Y.J., Xu X.R., Chen Y.M., Li H.B. (2014). Resources and biological activities of natural polyphenols. Nutrients.

[32] Halliwell B. (1990). How to characterize a biological antioxidant. Free Radic. Res. Commun..

[33] Heim K.E., Tagliaferro A.R., Bobilya D.J. (2002). Flavonoid antioxidants: Chemistry, metabolism and structure-activity relationships. J. Nutr. Biochem..

[34] Huttemann M., Lee I., Samavati L., Yu H., Doan J.W. (2007). Regulation of mitochondrial oxidative phosphorylation through cell signaling. Biochim. Biophys. Acta.

[35] Fukumoto L.R., Mazza G. (2000). Assessing antioxidant and prooxidant activities of phenolic compounds. J. Agric. Food Chem..

